# Do witnesses of functional/dissociative seizures recall them accurately? A UK experimental study of semiology recall at clinically relevant time points with a pilot intervention

**DOI:** 10.1002/epi.70388

**Published:** 2026-07-10

**Authors:** Adam John Noble, Steven Lane, Amina Al‐Khaddar, Annabelle Blundy, Brenna Dubois, Chloe Pearce, Laszlo Sztriha, Graham Powell, Alistair Wardrope, Rajiv Mohanraj, Anthony Guy Marson, Markus Reuber

**Affiliations:** ^1^ Department of Public Health, Policy, and Systems University of Liverpool Liverpool UK; ^2^ Department of Health Data Science University of Liverpool Liverpool UK; ^3^ Department of Psychological Sciences University of Liverpool Liverpool UK; ^4^ Department of Neurology King's College Hospital National Health Service Foundation Trust London UK; ^5^ Neurology Department Walton Centre National Health Service Foundation Trust Liverpool UK; ^6^ Department of Neuroscience School of Medicine and Population Health, University of Sheffield Sheffield UK; ^7^ Manchester Centre for Clinical Neurosciences, Salford Royal, Northern Care Alliance National Health Service Foundation Trust Salford UK; ^8^ Department of Pharmacology and Therapeutics University of Liverpool Liverpool UK

**Keywords:** accurate diagnosis, functional/dissociative seizure, recall, waiting times, witness

## Abstract

**Objective:**

Different causes of transient loss of consciousness (TLOC) carry distinct risks and require different management. Early accurate diagnosis is essential to guide counseling, investigation, and referral. In routine practice, frontline clinicians and specialists often rely on eyewitness accounts, yet the reliability of witness testimony for functional/dissociative seizures (FDSs) is unknown. We, for the first time, evaluated eyewitness recall of a single FDS immediately and after clinically relevant delays of 2 and 7 weeks, reflecting waiting times for specialist assessment in the United Kingdom (UK) and elsewhere. We also examined the relationship between confidence and accuracy, and whether early structured questioning improves later recall.

**Methods:**

In this UK‐based experimental study, general population adults (≥18) viewed a video of an FDS and were randomized to four conditions: A (immediate free recall + 2‐week follow‐up), B (immediate free recall + 7‐week follow‐up), C (immediate systematic questioning + 2‐week follow‐up), and D (immediate systematic questioning + 7‐week follow‐up). The primary outcome was accuracy on 15 standardized semiology questions, scored against consensus ratings from five neurologists.

**Results:**

Of 300 participants, 286 (95.3%) viewed the video in full; 90% completed follow‐up. Immediately afterward, participants answered only 67.0% of questions correctly. Accuracy declined to 58.8% at 2 weeks and was a further 5.4% lower at 7 weeks (95% confidence interval [CI] = 2.07–8.73). Features sometimes considered diagnostically informative—asynchronous limb movements, head movements, ictal eye closure—were prone to decay. Confidence was weakly associated with accuracy. Immediate systematic questioning did not improve follow‐up recall compared with free recall (−2.9, 95% CI = −6.3 to .5).

**Significance:**

Eyewitness recall of FDSs is modest and declines over clinically relevant intervals, and confidence poorly reflects accuracy. Early structured questioning did not improve retention. These findings highlight a key constraint on diagnostic accuracy in TLOC. Strategies to support differentiation in routine care are needed.

**Key points**

- Eyewitness accounts guide TLOC diagnosis, but reliability for FDSs has been untested until now.
- Immediate recall of FDSs is modest (~67%), even under optimal viewing conditions.
- Recall declines further over 2–7 weeks, reflecting real‐world delays to specialist review.
- Witness confidence poorly predicts accuracy, limiting its clinical value.
- An intervention that involved early systematic questioning did not appear to improve later recall.

## 1 INTRODUCTION

Transient loss of consciousness (TLOC)—a spontaneous, nontraumatic disturbance of consciousness with complete recovery^1^—affects approximately 50% of individuals at some point in their lives^2^ and frequently presents in urgent and emergency care settings.^3^ More than 90% of cases are attributable to epilepsy, functional/dissociative seizures (FDSs), or syncope,^4^ conditions that differ substantially in management and risk, making accurate early diagnosis essential.^5^, ^6^ The diagnostic gold standard is the capture of an event with concurrent physiological recording,^7^ but this is rarely achievable in routine practice. Moreover, most patients are asymptomatic and have no examination abnormalities at the time of assessment.^8^ Consequently, diagnoses—whether an initial working diagnosis made when the person first presents to a frontline service or a formal diagnosis that can follow subsequent specialist review—will often rely heavily on clinical history to reconstruct event semiology.

Eyewitness accounts can be pivotal to such reconstructions, because patients may have incomplete or distorted recall of the event. In specialist settings, particularly among patients with recurrent events, combining patient and witness reports has been shown to improve diagnostic accuracy.^9^, ^10^ Whereas individual semiological features are often nonspecific, combinations of features can aid differentiation between causes of TLOC.^9^, ^11^, ^12^, ^13^ However, the reliability of eyewitness recall following a first TLOC event—especially after clinically relevant delays—remains unclear.

Emerging evidence suggests that eyewitness recall of a single epileptic seizure is only moderately accurate and declines over time.^14^, ^15^, ^16^, ^17^ In our recent experimental study,^17^ general population observers of a focal‐to‐bilateral tonic–clonic seizure correctly answered a mean of only 62% of semiology‐related questions immediately after the event. These findings may help explain why up to one third of patients presenting with TLOC receive an initial diagnosis later judged incorrect by expert review.^18^ An accurate working diagnoses is important, given that it shapes immediate advice, investigations, and the care pathway that patients are referred to, with potential downstream consequences, including delays, if incorrect.^2^, ^19^ Our previous study also demonstrated that accurate recollection of details decreased to 54% at 2 weeks and 50% at 7 weeks. These intervals align with recommended^20^, ^21^ and real‐world waiting times for specialist assessment in the United Kingdom (UK) and elsewhere.^22^, ^23^, ^24^, ^25^, ^26^, ^27^ The study also identified which features were most reliably recalled and showed that witness confidence poorly predicted accuracy.

Whether our findings regarding moderate witness accuracy generalize to other causes of TLOC, particularly FDSs, remains unknown. A systematic literature search identified no relevant evidence addressing this question (Appendix S1). Several factors could plausibly cause eyewitness recall to differ for a single epileptic seizure and FDS, although the direction of any effect remains uncertain. Epileptic seizures—particularly tonic–clonic ones, which commonly prompt health care contact—often involve salient, stereotyped features such as sudden collapse, rhythmic movements, and postictal confusion. These characteristics may support encoding and later recall. By contrast, FDSs are typically longer in duration (median = 3.4 min vs. 1.5 min for focal‐to‐bilateral tonic–clonic seizures)^28^ and less rhythmic, with asynchronous limb movements, side‐to‐side head movements, and ictal eye closure suggestive of FDS.^13^ Such differences may affect how attention is allocated during the event and how memories are subsequently reconstructed. Witness expectations may also influence recall. Public understanding of “seizures” appears to be shaped largely by a tonic–clonic prototype.^29^, ^30^ In the absence of a clear schema for FDS, witnesses may default to familiar descriptors, potentially distorting recall. Conversely, weaker preexisting expectations might reduce schema‐driven intrusions and promote closer reliance on observed features. These competing possibilities highlight the need to examine eyewitness recall of FDSs.

It is also important to identify opportunities to optimize witness recall, even if modestly, with a view to supporting improved diagnostic accuracy. In patients with chronic, recurrent TLOC episodes, misdiagnosis rates range from 20% to 30%.^31^, ^32^ For FDSs, the mean delay from initial presentation to correct diagnosis is approximately 7 years, during which patients may undergo unnecessary investigations, receive inappropriate treatments, and experience iatrogenic harm, while lacking access to appropriate psychological interventions.^33^

Preliminary evidence from Noble et al.^17^ suggests that recall of a single epileptic seizure may be enhanced through early, structured questioning. This has potential practical implications for first responders, such as paramedics, who are often the first health care professionals to encounter witnesses, but who may obtain only informal accounts at the scene, with more structured histories only elicited later. Specifically, they demonstrated that prompting participants to systematically report observed features shortly after the event improved recall accuracy at 2 and 7 weeks by approximately 7% compared with unstructured free recall. This is consistent with findings from forensic psychology indicating that structured recall within 24 h may reduce memory decay and susceptibility to misinformation.^34^ Whether similar benefits extend to FDSs remains unknown, and pilot data are required to inform a definitive trial.

This experimental study thus aimed to address the aforementioned evidence gaps by (1) evaluating, for the first time, the accuracy of eyewitness recall of an FDS immediately after observation and at 2 and 7 weeks; (2) assessing the relationship between witness confidence and recall accuracy; and (3) exploring whether systematic postevent questioning might improve longer term recall compared with free recall, and its implications for a definitive trial.

## 2 MATERIALS AND METHODS

### 2.1 Design

The experiment was conducted via the online platform Qualtrics, where participants remotely viewed items and responded to questionnaires at different assessment points. A 2 × 2 factorial between‐subjects design was employed. The independent variables were the following: (1) time between stimulus video and final recall (2 weeks vs. 7 weeks) and (2) immediate management to secure initial recall (“free recall” vs. “systematic questions”). Participants were randomly assigned to one of four conditions (A, B, C, D) in a 1:1:1:1 ratio. The dependent variable was accuracy of witness recall.

### 2.2 Ethics

The University of Liverpool's Institute of Population Health Research Ethics Committee approved the study (Ref: 17266). All participants provided informed consent.

### 2.3 Recruitment

Participants were recruited in January 2026 via Prolific, an online platform that maintains a panel of individuals who have registered interest in participating in research studies. Eligible participants were adults residing in the UK. Invitations were distributed by Prolific to a stratified sample based on age, sex, and ethnicity to approximate the UK general adult population, in line with census distributions.

Participants were informed that the study involved eyewitness accounts but were not told its specific aims or hypotheses. They received £7 for completing the baseline questionnaire and £2.50 for the follow‐up. Further recruitment details are provided in Appendix S2.

### 2.4 Eligibility criteria

Participants were UK residents aged ≥18 years, able to complete a survey in English, with normal or corrected vision and hearing, and internet access with audio. Exclusion criteria included terminal illness, severe psychiatric conditions, and an inability to provide informed consent. Prolific did not invite persons who participated in our prior experiment.^17^

### 2.5 Measures

#### 2.5.1 Witness recall

Recall of the observed event was assessed at baseline (conditions C, D) and follow‐up (all conditions) using 15 items from Erba et al.'s^35^, ^36^ suspected seizure witness questionnaire (Table 1). Items focused on seizure semiology; 14 items were the same as those used in the prior study with an epileptic seizure video. Five UK consultant neurologists with seizure expertise^37^ reviewed the video and completed the same items to provide a consensus‐based judgment against which participants' answers were compared (Appendix S3). Across items, median modal agreement among neurologists was 80% (interquartile range [IQR] = 60–100).

**TABLE 1:** Items used to assess participant recall and the accuracy of the recall of key groups.

Overall recall accuracy	Comparisons of interest									
	Recall accuracy when participants are grouped by time of assessment						Recall accuracy at follow‐up when participants are grouped by immediate management			
	Immediate assessment, conditions C + D, *n* = 150		2‐week follow‐up assessment, conditions A + C, *n* = 137		7‐week follow‐up assessment, conditions B + D, *n* = 132		Free recall, conditions A + B, *n* = 135		Systematic recall, conditions C + D, *n* = 134	
% correct										
Mean (SD)	67.0 (12.9)		58.8 (12.5)		53.4 (15.2)		57.6 (13.9)		54.7 (14.2)	
Median (IQR)	66.7 (60.0–73.3)		60.0 (53.3–66.7)		53.3 (46.7–66.7)		60.0 (46.7–66.7)		53.3 (46.7–66.7)	
Minimum–maximum	13.3–93.3		13.3–86.7		13.3–93.3		13.3–86.7		13.3–93.3	
Raw										
Mean (SD)	9.9 (1.9)		8.8 (1.9)		8.0 (2.3)		8.6 (2.9)		8.2 (2.1)	
Median (IQR)	10.0 (9.0–11.0)		9.0 (8.0–10.0)		8.0 (7.0–10.0)		9.0 (7.0–10.0)		8.0 (7.0–10.0)	
Minimum–maximum	2.0–14.0		2.0–13.0		2.0–14.0		2.0–13.0		2.0–14.0	

Individual recall items	Correct, *n* (%)	Rank^a^	Correct, *n* (%)	Rank^a^	Correct, *n* (%)	Rank^a^	Correct, *n* (%)	Rank^a^	Correct, *n* (%)	Rank^a^
1. Did you observe any of the following at the very beginning of the seizure? *Correct answer: “None of these”*	90 (60.0)	10	80 (58.4)	7	71 (53.8)	8	86 (63.7)	7	65 (48.5)	9
2. Did the patient shake or stiffen? *Correct answer: “Yes”*	148 (98.7)	1	133 (97.1)	=1	123 (93.2)	1	128 (94.8)	1	128 (95.5)	=1
3. How did the patient shake or stiffen? *Correct answer: “On both sides equally”*	99 (66.0)	8	66 (48.2)	10	56 (42.4)	9	68 (50.4)	9	54 (40.3)	10
4. Did the shaking or stiffening stop abruptly and then start back up during the seizure? *Correct answer: “Yes”*	103 (68.7)	7	79 (57.7)	=8	74 (56.1)	7	84 (62.2)	8	69 (51.5)	=7
5. How did the shaking or stiffening stop? *Correct answer: “Slowed down gradually”*	120 (80.0)	6	106 (77.4)	5	99 (75.0)	5	97 (71.9)	5	108 (80.6)	4
6. Did the patient's head turn strongly to one side? *Correct answer: “No”*	56 (37.3)	14	58 (42.3)	11	28 (21.2)	14	50 (37.0)	12	36 (26.9)	14
7. Was there movement of the head from side to side? *Correct answer: “Yes”*	88 (58.7)	11	48 (35.0)	13	41 (31.1)	12	40 (29.6)	13	49 (36.6)	12
8. During the seizure, were the patient's eyes closed or open? *Correct answer: “Closed”*	127 (84.7)	5	79 (57.7)	=8	54 (40.9)	10	64 (47.4)	10	69 (51.5)	=7
9. Did you see any of the following during the seizure? *Correct answer: “None of these”*	8 (5.3)	15	16 (11.7)	15	13 (9.8)	15	19 (14.1)	15	10 (7.5)	15
10. Do you see any of the following during the seizure? *Correct answer: “Limb movements that were ‘out of sync’”*	67 (44.7)	12	53 (38.7)	12	48 (36.4)	11	51 (37.8)	11	50 (37.3)	11
11. How was the patient's breathing during the seizure? *Correct answer: “Normal”*	58 (38.7)	13	44 (32.1)	14	39 (29.5)	13	39 (28.9)	14	44 (32.8)	13
12. Did the patient fall during the seizure? *Correct answer: “No”*	147 (98.0)	2	133 (97.1)	=1	119 (90.2)	2	124 (91.9)	2	128 (95.5)	=1
13. How long did the actual seizure activity last? *Correct answer: “2–10 min”*	141 (94.0)	3	111 (81.0)	4	101 (76.5)	4	107 (79.3)	4	105 (78.4)	5
14. Right at the end of the seizure activity, did you see any of the following? *Correct answer: “None of these”*	95 (63.3)	9	83 (60.6)	6	80 (60.6)	6	94 (69.6)	6	69 (51.5)	=7
15. Right at the end of the seizure activity, what was their level of consciousness and awareness? *Correct answer: “Awake but confused and disoriented”*	138 (92.0)	4	119 (86.9)	3	111 (84.1)	2	115 (85.2)	3	115 (85.8)	3

^a^Questions for each recall item were ranked for each condition/assessment point according to the proportion of correct responses elicited (with rank 1 indicating a question with the highest proportion of correct responses; within the rankings, an equal sign [=] indicates a joint rank).

Abbreviation: IQR, interquartile range.

#### 2.5.2 Confidence

At follow‐up, participants rated their confidence in their recall on a 1–9 Likert scale (1 = “pure guess,” 9 = “absolutely certain”) in response to: “How confident are you in your answers about the seizure in the video?” Within forensic psychology research, confidence is typically assessed using numerical rating scales. We elected to use the same question as Noble et al.,^17^ because there is no agreed, validated measure of recall confidence^38^ and because it did not involve repeatedly interrupting participants during their memory retrieval process.^39^ Its 9‐point format additionally aligns with GRADE (Grading of Recommendations Assessment, Development, and Evaluation) recommendations for assessing judgment strengths.^40^

### 2.6 Procedure

At baseline, participants ensured their device sound was on and provided demographic information. They were then shown a 403‐s home video of a 50‐year‐old female diagnosed with FDSs, laying on her side on a sofa and experiencing an FDS event characterized by a pattern of “convulsive” or “thrashing” activity. Her case has previously been described in detail by Dickson et al.^41^ The diagnosis, made by author Markus Reuber, followed comprehensive clinical assessment including continuous 48‐h video telemetry during which a typical event was captured.

Although awareness or responsiveness were not formally assessed during the recorded event, the patient appeared unresponsive throughout much of the seizure. The episode involved coarse tremulous limb movements of variable frequency and amplitude, together with side‐to‐side head movements. Saliva was visible at the angle of the mouth, and the patient's eyes remained persistently closed during the shaking. The tremulous movements gradually subsided after approximately ~2.5 min, and the patient's eyes opened after ~3 min. Between 4.5 and 6 min, her eyes closed again and gentler limb shaking movements started. Near the end of the recording (after ~360 s), the patient appeared to respond when spoken to. The video was shown in its original form, and the patient's face was fully visible.

Although no formal validation study of the video was conducted, it was selected because it was considered to depict a common pattern for persons with FDSs, along with “swoon,” “catatonic,” or “pseudosyncope” events.^7^ The panel of neurologists who reviewed the video and established the reference standard against which participants' recall accuracy was assessed excluded MR due to his prior familiarity with the case.

Afterward, participants completed a 9‐min filler task designed to simulate ambulance response time in European nations (e.g., Noble et al.^42^) and thus the typical time between witnessing a suspected seizure and first being asked to recall it in any detail. The task set included an attention check (“…was the person in the video who had the seizurelike event outdoors?” Yes/No), questions about the device used, and then simple distractor questions.^43^ Following this, participants were randomized into one of four conditions. Conditions A and B asked for free recall descriptions; conditions C and D involved answering the 15 systematic questions from Erba et al.'s^35^ questionnaire. Participants were then asked about personal/familial seizure experience.

Participants were told they would complete a follow‐up after 2 weeks (conditions A, C) or 7 weeks (conditions B, D). A single reminder was sent to persons to complete their follow‐up questionnaire.

### 2.7 Analysis

The sample size was based on estimating key parameters for a future trial. A target of 50 per condition was deemed sufficient to estimate variation and effect, increased by 33% to allow for missing data and possible drop‐outs, yielding 65 per condition. Previous recommendations suggest 24–50 participants are adequate for pilot studies.

Analyses were conducted using Stata (v18.0). Descriptive statistics summarized baseline characteristics and compared (without formal statistical tests) participants' age, sex, and ethnicity with UK census data.

Recall accuracy—defined as the proportion of correctly answered questions—was calculated for conditions C and D at baseline, and for all conditions at follow‐up. The effect of time was estimated by using independent‐samples *t‐*tests to compare follow‐up recall between the 2‐week conditions (A + C) and the 7‐week conditions (B + D), and paired‐samples *t‐*tests comparing immediate recall in conditions C and D with recall at their respective follow‐ups. The effect of immediate management was estimated by using independent samples *t*‐tests to compare free recall conditions (A + B) with systematic recall conditions (C + D). Mean differences along with 95% confidence intervals (CIs) are presented. Analyses of clinical outcomes were exploratory, as the pilot study was not informed by a formal power calculation. Accordingly, *p*‐values should be interpreted cautiously. All tests were two‐sided, and independent‐samples *t*‐tests assuming unequal variances were used throughout.

Recall questions were ranked by the proportion of correct responses across conditions. Accuracy is also separately reported for three potentially key items from the full questionnaire: Q7 (side‐to‐side head movements), Q8 (ictal eye closure), and Q10 (limb movements that were “out of sync”).^13^

Spearman rank correlations assessed the relationship between recall accuracy and confidence.

A priori, it was specified that if the mean difference in recall between the systematic recall condition (C + D) and the free recall condition (A + B) exceeded zero in favor of the intervention, a sample size for a definitive trial would be estimated. Using a two‐sided *α* of .05 and the variability in follow‐up recall observed in this study, G*Power (v3.1.9.7) would calculate the sample size required to achieve 90% power to detect an effect corresponding to the lower bound of the 95% CI for the mean difference between conditions A + B and C + D.

## 3 RESULTS

### 3.1 Participant recruitment, allocation, watching of stimulus video, and completion time

Of 311 invited individuals, 306 consented to participate. Of these, 300 were randomized (Appendix S4): 75 to condition A, 75 to condition B, 75 to condition C, and 75 to condition D.

Nearly all participants (*n* = 286, 95.3%) watched the video in full, with *n* = 298, (99.3%) passing an attention check (Table 2). There were no obvious differences between conditions in the proportions of participants fully watching the video or distractor task engagement.

**TABLE 2:** Characteristics of all participants recruited, by randomized condition and in comparison to UK adult population.

Characteristic	Consented, but not randomized, *n* = 6	Consented & completed baseline, *n* = 300	If UK representative, *n* = 300^a^	Condition			
				A, *n* = 75	B, *n* = 75	C, *n* = 75	D, *n* = 75
Age, years							
Median (IQR)	58 (23.5–63.0)	48 (33–58)	‐	48 (33–59)	48 (32–59)	47 (29–58)	48 (36–58)
Range	21–65	19–80	‐	20–80	19–73	20–71	20–75
Missing	1	0	‐	0	0	0	0
18–24, *n* (%)	1 (20.0)	33 (11.0)	32 (10.7)	9 (12.0)	8 (10.7)	10 (13.3)	6 (8.0)
25–34, *n* (%)	1 (20.0)	50 (16.7)	50 (16.7)	12 (16.0)	14 (18.7)	13 (17.3)	11 (14.7)
35–44, *n* (%)	3 (60.0)	47 (15.7)	48 (16.0)	13 (17.3)	11 (14.7)	10 (13.3)	13 (17.3)
45–54, *n* (%)	0	53 (17.7)	51 (17.0)	10 (13.3)	14 (18.7)	13 (17.3)	16 (21.3)
55–100, *n* (%)	0	117 (39.0)	119 (39.7)	31 (41.3)	28 (37.3)	29 (38.7)	29 (38.7)
Missing, *n*	1	0	0	0	0	0	0
Sex, *n* (%)							
Female	1 (20.0)	155 (51.7)	156 (52.0)	40 (53.3)	37 (49.3)	38 (50.7)	40 (53.3)
Male	4 (80.0)	144 (48.0)	144 (48.0)	35 (46.7)	37 (49.3)	37 (49.3)	35 (46.7)
Prefer not to say	0	1 (.2)	0	0 (0)	1 (1.3)	0	0
Missing	1	0	0	0	0	0	0
Ethnicity							
White	‐	247 (82.3)	247 (82.3)	62 (82.7)	65 (86.7)	59 (78.7)	61 (81.3)
Asian	‐	23 (7.7)	23 (7.7)	4 (5.3)	4 (5.3)	8 (10.7)	7 (9.3)
Black	‐	10 (3.3)	10 (3.3)	3 (4.0)	3 (4.0)	3 (4.0)	1 (1.3)
Mixed	‐	10 (3.3)	10 (3.3)	3 (4.0)	1 (1.3)	5 (6.7)	1 (1.3)
Other	‐	10 (3.3)	10 (3.3)	3 (4.0)	2 (2.7)	0 (0)	5 (6.7)
Missing	6	0	‐	0	0	0	0
English first language, *n* (%)^b^							
Yes	‐	272 (90.7)	271 (90.3)	66 (88.0)	69 (92.0)	68 (90.7)	69 (92.0)
Missing	6	0	‐	0	0	0	0
Seizure experience, *n* (%)							
Experienced seizure &/or dx. seizure disorder	‐	6 (2.0)	‐	3 (4.0)	1 (1.3)	2 (2.7)	0 (0)
Missing	6	0	‐	0	0	0	0
Family/friend seizure &/or dx. seizure disorder	‐	83 (27.7)	‐	22 (29.3)	30 (40.0)	17 (22.7)	14 (18.7)
Missing	6	0	‐	0	0	0	0
Seen someone have a suspected seizure in person	‐	116 (38.7)	‐	32 (42.7)	35 (46.7)	25 (33.3)	24 (32.0)
Missing	6	0	‐	0	0	0	2
Watched video completely, *n* (%)							
Yes	‐	286 (95.3)	‐	70 (93.3)	73 (97.3)	71 (94.7)	72 (96.0)
Missing	‐	0	‐	0	0	0	0
Device watched on, *n* (%)							
Phone	‐	28 (9.3)	‐	3 (4.0)	7 (9.3)	8 (10.7)	10 (13.3)
Tablet	‐	16 (5.3)	‐	5 (6.7)	3 (4.0)	2 (2.7)	6 (8.0)
Computer	‐	256 (85.3)	‐	67 (89.3)	65 (86.7)	65 (86.7)	59 (78.7)
Missing	‐	0	‐	0	0	0	0
Attention check, *n* (%)							
Passed	‐	298 (98.0)	‐	74 (98.7)	74 (98.7)	75 (100.0)	75 (100.0)
Missing	‐	0	‐	0	0	0	0
Distractor questions answered, *n*/137							
Median (IQR)	‐	53.5 (39–77)	‐	45 (39–61)	60 (42–89)	50 (39–75)	57 (40–85)
Range	‐	0–137	‐	0–130	26–137	18–137	7–128
Answered all questions, *n* (%)	‐	4 (1.3)	‐	0 (0)	3 (4.0)	1 (1.3)	0 (0)
Missing		0	‐	0	0	0	0
Answered no questions, *n* (%)		1 (.3)	‐	1 (1.3)	0 (0)	0 (0)	0 (0)
Missing		0	‐	0	0	0	0
Free recall engagement^c^							
Median number of words used (IQR)	‐	50 (32.8–87.3)	‐	56 (33–84)	46 (32–91)	‐	‐
Range	‐	7–305	‐	9–305	7–201	‐	‐
Missing	‐	0	‐	0	0	‐	‐

*Note*: Condition A, immediate free recall, 2‐week follow‐up; condition B, immediate free recall, 7‐week follow‐up; condition C, immediate systematic recall, 2‐week follow‐up; condition D, immediate systematic recall, 7‐week follow‐up.

Abbreviations: dx., diagnosed; IQR, interquartile range.

^a^ Age, sex, and ethnicity estimates are derived from the 2021/2022 Census data that reflect England and Wales only.

^b^ The question within the Census was not exactly the same as asked in the study. It was instead, “What is your main language?”.

^c^ Task only requested of the participants in conditions A and B.

The median time it took participants to complete the baseline questionnaire was similar for those for whom it involved free recall (19.9 min, IQR = 18.5–22.5) and for those for whom it involved responding to the systematic questions (19.9 min, IQR = 18.4–22.2). Participants in conditions A + B typed a median of 50 words (IQR = 32.8–87.3) in response to the free recall question.

### 3.2 Participant demographics

Median participant age was 48.0 years (IQR = 33–58, range = 19–80); 155 (51.7%) were female (Table 2). Participants were comparable in age, sex, first language, and ethnicity to the UK adult population, and participants in the different conditions were broadly similar in demographics and previous seizure experiences. Of the participants, 116 (38.7%) reported having witnessed a suspected seizure in person (condition A, 42.7%; condition B, 46.7%; condition C, 33.3%; condition D, 32.0%).

### 3.3 Participant retention

Two hundred sixty‐nine (89.7%) participants returned their follow‐up questionnaire with complete responses to the 15 items testing their recall. It took them a median of 3.4 min (IQR = 2.6–4.9) to complete. Retention was minimally higher in conditions with a 2‐week follow‐up (A + C, 91.3%) compared to those with a 7‐week follow‐up (B + D, 88.0%; Appendix S4).

### 3.4 Accuracy of witness descriptions, variation, and confidence

#### 3.4.1 Effect of scheduled follow‐up time

##### Accuracy of recall at immediate assessment and at 2‐ and 7‐week follow‐up

Participants asked systematic questions (conditions C + D) immediately after watching the video answered a mean of 67.0% (SD = 12.9) of them correctly (Table 1, Figure 1A). These participants answered significantly fewer questions correctly at their 2‐ or 7‐week follow‐up (Appendix S5 presents accuracy for each condition). The largest difference between immediate and follow‐up recall was seen for condition D (7‐week follow‐up), with a mean difference in the proportion answered correctly of −13.0 (95% CI = −16.6 to −9.5, *t*[67] = 7.28, *p* < .001). For participants in condition C (2‐week follow‐up), the mean difference was −8.8 (95% CI = −11.9 to −5.6, *t*[65] = 5.48, *p* < .001).

**FIGURE 1:**
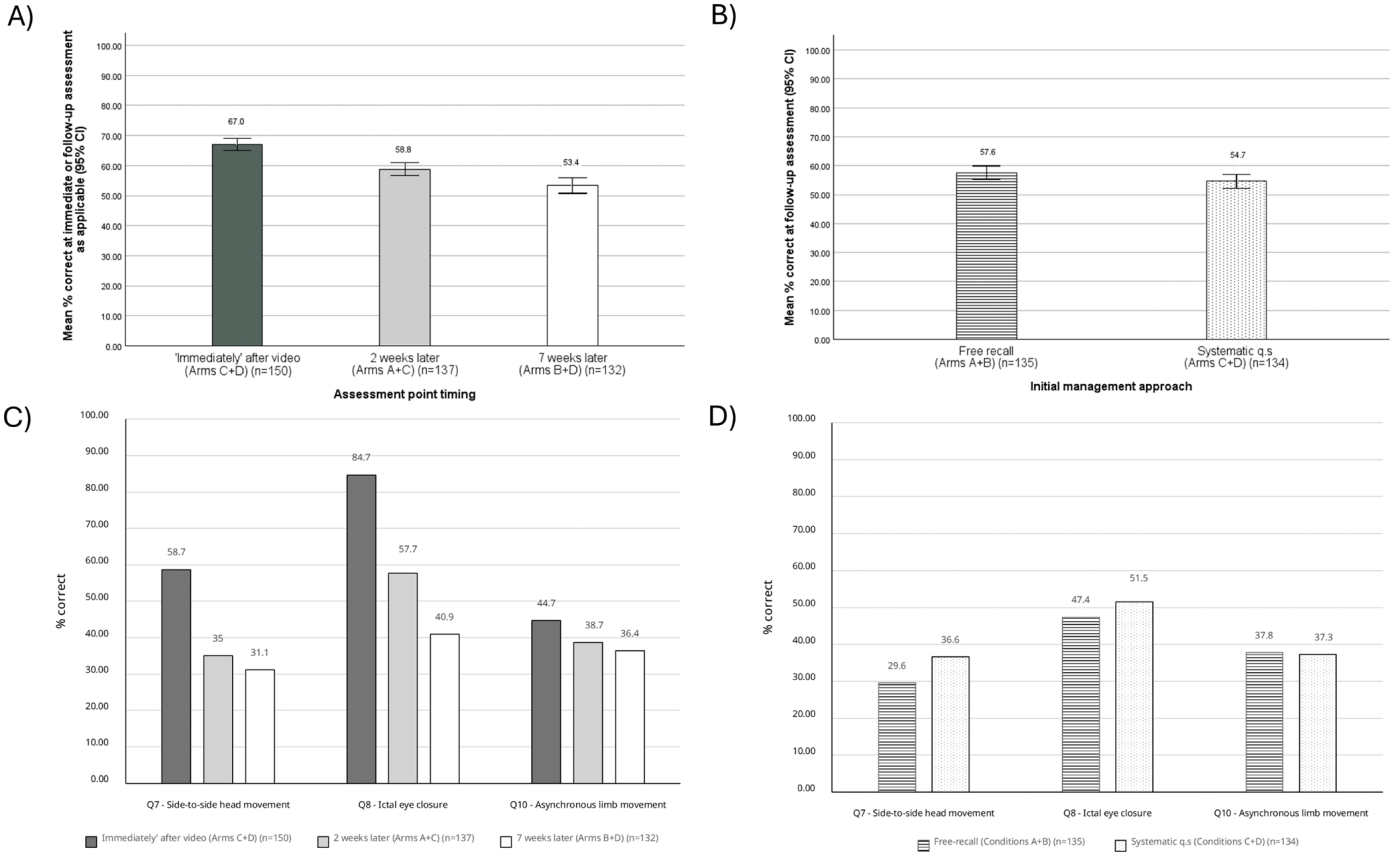
(A) Mean proportion of questions answered correctly by participants groups immediately afterward and at 2‐ and 7‐week follow‐up. (B) Percentage of participant groups immediately afterward and at 2‐ and 7‐week follow‐up that correctly answered questions about specific seizure features (head movement, eye closure, and limb movement). (C) Mean proportion of questions answered correctly by participant groups at 2‐ and 7‐week follow‐up according to whether the person received immediate systematic questioning. (D) Percentage of participants groups at follow‐up according to whether the person received immediate systematic questioning that correctly answered questions about specific seizure features (head movement, eye closure, and limb movement). CI, confidence interval; q.s, questioning. Condition A, immediate free recall, 2‐week follow‐up; condition B, immediate free recall, 7‐week follow‐up; condition C, immediate systematic recall, 2‐week follow‐up; condition D, immediate systematic recall, 7‐week follow‐up.

In line with this, participants in the 2‐week follow‐up conditions (A + C) answered significantly more questions correctly at follow‐up (mean = 58.8, SD = 12.5) than participants in the 7‐week follow‐up conditions (B + D; mean = 53.4, SD = 15.2; mean difference = 5.4, 95% CI = 2.1–8.7, *t*[253.5] = 3.19, *p =* .002; Figure 1A). In absolute terms, this difference was, however, small. It equated to participants answering a mean of .81 (95% CI = .31–1.31, *t*[253.5] = 3.18, *p* = .002) more questions correctly (Table 1, Appendix S5).

The items answered with the lowest and highest accuracy (Q9 and Q2, respectively) were consistent across all time points (i.e., at the immediate assessment and at 2‐ and 7‐week follow‐ups; Table 1). Participants showed the highest accuracy for gross motor features, event duration, and consciousness level, and lowest recall for behavioral and motor semiology and breathing (Figure 2). Accuracy for the potentially key semiological features—head movement (Q7), ictal eye closure (Q8), and asynchronous limb movements (Q10)—was highest immediately after video observation, declined at 2 weeks, and decreased further at 7 weeks (Figure 1C). For instance, 58.7% of participants answered the question regarding side‐to‐side head movement correctly at the immediate assessment, compared to 35.0% at the 2‐week follow‐up and 31.1% at 7 weeks.

**FIGURE 2:**
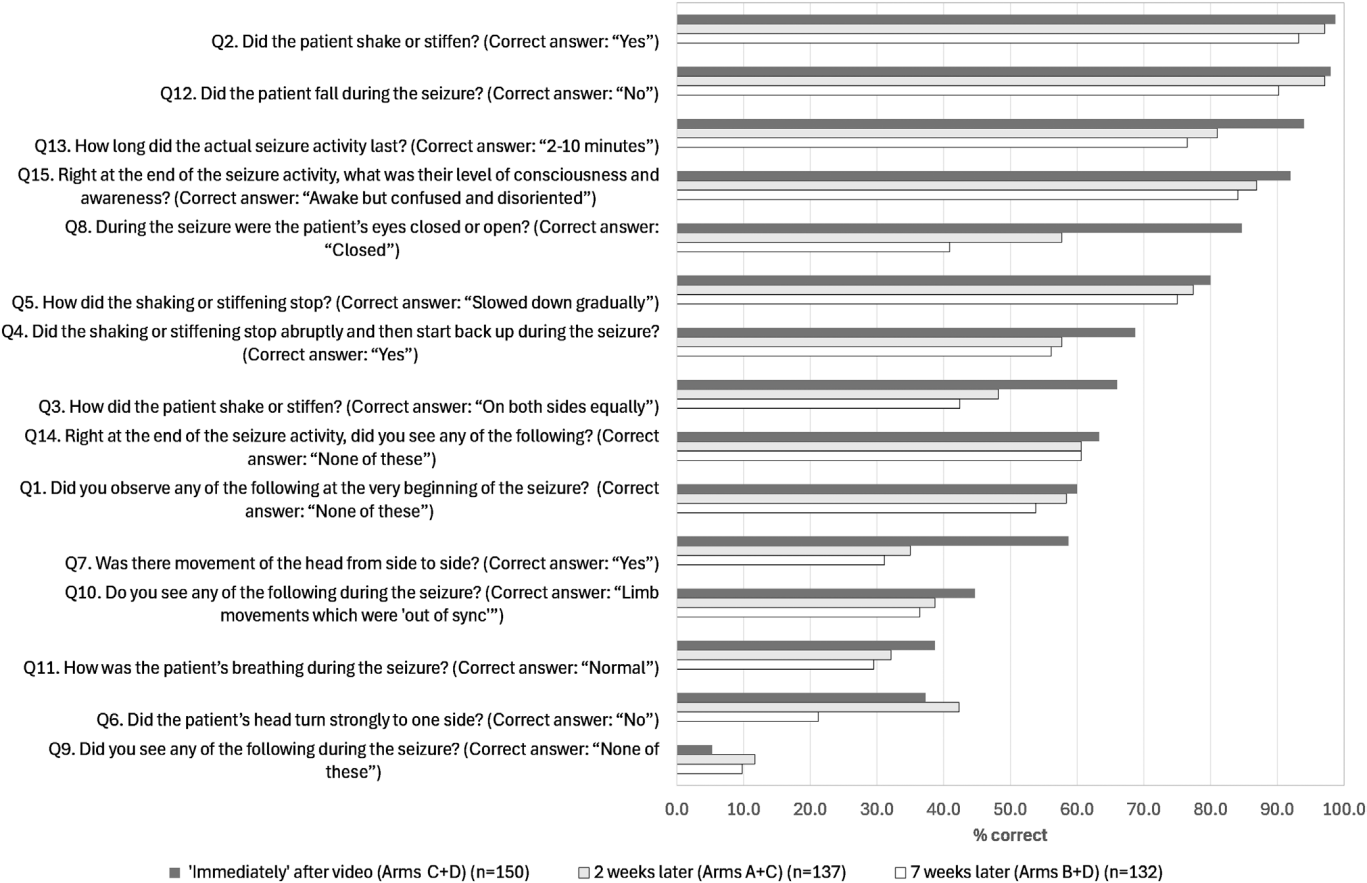
The percentage of participant groups immediately afterward and at 2‐ and 7‐week follow‐up that correctly answered the different individual questions about specific seizure features. Condition A, immediate free recall, 2‐week follow‐up; condition B, immediate free recall, follow‐up; condition C, immediate systematic recall, 2‐week follow‐up; condition D, immediate systematic recall, 7‐week follow‐up. Questions are ordered by the proportion of participants in conditions C + D who answered them correctly “immediately” after they viewed the video.

##### Participants' confidence in their recall at follow‐up and association with accuracy

Participants followed up at 2 weeks expressed slightly higher confidence in their recall (mean = 5.8, SD = 1.3) than those followed up at 7 weeks (mean = 4.9, SD = 1.6). Among participants followed up at 2 weeks, confidence held only a small positive association with recall accuracy (*ρ*[137] = .16). Confidence was slightly more moderately associated with accuracy for participants followed up at 7 weeks (*ρ*[(132)] = .33).

#### 3.4.2 Effect of immediate management to secure initial recall

Participants who were initially asked systematic questions (conditions C + D) did not answer more questions correctly at follow‐up than participants who were not (Figure 1B). They answered a mean of 54.7% (SD = 14.2) of questions correctly compared to 57.6% (SD = 13.1) by those asked to complete free recall (conditions A + B; mean difference = −2.9, 95% CI = −6.3 to .5, *t*[266.8] = 1.694, *p* = .09). This equated to them answering a mean of −.4 (95% CI = −.9 to .1, *t*[266.86 = 1.694, *p* = .09) fewer questions correctly. Both groups were least and most accurate on the same items (Table 1).

Condition C + D participants did, however, outperform condition A + B participants on the questions related to side‐to‐side head movements (Q7, 36.6% correct vs. 29.6%) and eye closure (Q8, 51.5% vs. 47.4%), but not asynchronous limb movement (Q10, 37.3% vs. 37.8%; Figure 1D).

## 4 DISCUSSION

In this experimental study, we, for the first time, examined the accuracy of eyewitness recall of a single FDS at clinically and internationally relevant time intervals. Three principal findings emerge. First, recall accuracy was modest even immediately after observation and declined further over time, although the magnitude of this decline was relatively small. Second, there was no evidence that early structured questioning improved later recall. Third, witness confidence was only weakly associated with accuracy. Together, these findings have implications for clinical practice and for research aimed at improving diagnostic pathways for TLOC.

### 4.1 Recall accuracy, temporal decline, and confidence

Eyewitness recall of the FDS was limited from the outset. Participants correctly reported approximately two thirds of semiological features immediately after observation, declining to ~59% at 2 weeks and ~53% at 7 weeks. Although the absolute reduction was modest, this pattern is clinically relevant, as these intervals reflect typical delays before specialist assessment following a first TLOC episode.^22^, ^23^, ^24^, ^25^, ^26^, ^27^

These findings closely align with our prior work on eyewitness recall of a single epileptic seizure,^17^ where initial accuracy was similarly modest and declined at comparable rates (62% of questions answered correctly immediately afterward, 54% at 2 weeks, and 50% at 7 weeks). Despite differences in phenomenology between FDSs and epileptic seizures, recall performance appears broadly similar. This suggests that such differences do not meaningfully influence eyewitness memory, which appears constrained and similarly vulnerable to decay across event types.

The temporal pattern observed is consistent with established psychological theory, particularly the work of Ebbinghaus.^44^ His classic “forgetting curve” describes a rapid initial decline in memory followed by a slower rate of forgetting. Our findings mirror this trajectory, with the largest reduction occurring between immediate assessment and 2 weeks, followed by a smaller decline at 7 weeks. This concordance supports the generalizability of our findings and indicates that recall of medical events is subject to fundamental memory processes governing episodic memory.

Features sometimes considered diagnostically informative for FDSs were particularly susceptible to decay.^13^ Accurate reporting of ictal eye closure declined from 85% immediately postevent to 58% at 2 weeks and 41% at 7 weeks. Side‐to‐side head movements showed a similar pattern (59%, 35%, and 31%, respectively). Recall of asynchronous limb movements was consistently low (45%, 39%, and 36%). The limited accuracy—particularly for asynchronous limb movements, even immediately—highlights the need for caution when relying on eyewitness descriptions of these features in any diagnostic decision‐making.

We observed only weak correlations between witness confidence and recall accuracy, particularly at earlier time points. This is consistent with extensive evidence from forensic settings^45^ demonstrating that confidence is an unreliable indicator of memory accuracy and mirrors our previous findings in epileptic seizure recall.^17^

### 4.2 Effect of early structured questioning

We found no evidence that early structured questioning—which could have been an intervention adopted by paramedics to help better equip subsequent clinicians to make accurate diagnoses—improved recall at follow‐up. Participants who completed structured questioning shortly after observing the event did not demonstrate better retention than those providing free recall; if anything, estimates favored free recall, although with uncertainty.

Based on these pilot data, a definitive trial of this intervention may not currently be justified, although this interpretation should be tempered by the study's limitations and parameters, which may have influenced the findings. This contrasts with our prior study of epileptic seizure recall, in which early structured questioning was associated with a modest improvement in retention.^17^ One explanation is that the intervention may be more effective for tonic–clonic seizures, which have a narrower range of manifestations and shorter duration.

Alternatively, the video stimulus used in the present study may have elicited greater stress in participants. This is pertinent given evidence for an inverted U‐shaped relationship between stress and memory, whereby moderate stress enhances memory, whereas both low and high stress impair it.^46^ Participants in both studies were recruited via research platforms and viewed videos of seizures involving unfamiliar individuals, potentially limiting emotional engagement.^47^ However, emerging evidence now suggests viewing FDSs may be more distressing than viewing epileptic seizures.^48^ This increased distress may, in line with the aforementioned relationship, have enhanced participant recall and reduced the opportunity of benefit of structured questioning.

### 4.3 Clinical and research implications

Several practical implications arise. First, even under optimal conditions—controlled observation and immediate assessment—recall accuracy was modest. Clinicians should therefore be cautious in placing substantial weight on eyewitness accounts of a single FDS, particularly when there has been a delay between the event and assessment. We do acknowledge, however, that in specialist practice, formal diagnoses are typically made after more than a single event. In settings such as the UK, where waiting times to see a seizure specialist can be approximately 7 weeks, a significant minority of patients will also experience a further event—often witnessed—before assessment.^22^ Repeated events and multiple witness accounts may partially mitigate limitations in recall of a single event, although variability in accuracy remains an important consideration.

Second, witness confidence should not be used as a proxy for reliability. Highly confident accounts are not necessarily more accurate, and low‐confidence reports should not be dismissed. Evidence from the forensic literature indicates that confident witnesses are often perceived as more credible.^38^

Third, our findings support early capture of eyewitness accounts, ideally at first contact with health care services. In the UK, first‐responder documentation of suspected seizures is variable in detail and completeness. Given the rapid early decline in recall, timely documentation—particularly by paramedics—may be important. However, efforts to enhance early documentation should be balanced against our finding that structured questioning, as implemented here, does not improve recall and may have a small adverse effect.

Fourth, it is important to consider that judging whether certain semiological features are present is not straightforward and can involve some subjectivity. Consistent with this, neurologist raters in our study did not achieve complete agreement on all features. Our participants did not appear to struggle more with features that showed greater expert disagreement. Nevertheless, this observation—also seen in our prior work on epileptic seizures—highlights inherent limits on the accuracy of eyewitness reports and underscores the need to manage expectations accordingly.

Finally, given the ongoing challenges in differentiating causes of TLOC—particularly FDS—additional approaches to improve diagnostic accuracy are needed. One promising avenue is the structured analysis of patient accounts. Schwabe et al.^49^ identified interactional differences in how individuals with FDSs describe events compared with other causes of TLOC. Building on this, Pevy et al.^50^ generated a “digital doctor” tool that could theoretically be used by first responders to capture real‐time patient accounts and complete automated linguistic analysis to support diagnostic decision‐making.

### 4.4 Strengths and limitations

This study has several strengths. The sample was broadly representative of the UK adult general population in age, sex, and ethnicity, enhancing generalizability. The relatively large sample size and good retention at follow‐up provided estimates with narrow CIs. The use of standardized questions and a consensus‐derived gold standard from five consultant neurologists provided a robust benchmark for assessing recall accuracy. The study was also conducted and reported in accordance with guidelines.^51^

Limitations should also be considered. Ecological validity is unclear with participants—as a condition of ethical approval—being informed in advance about the nature of the study. Evidence suggests this is, however, unlikely to have substantially affected recall.^52^ Participants also viewed a video of a stranger. The emotional intensity of viewing the seizure was thus likely lower for our participants than for witnesses seeing a seizure in a known person.^47^ It is unclear how this might have impacted recall. Stress does affect memory, but the relationship, as noted earlier, is complex, with Gering et al.^46^ indicating too little or too much stress may impair memory. To avoid unintentionally improving recall, we thus did not seek to use experimental techniques to induce additional stress.

Findings are also based on a single video stimulus and therefore reflect the characteristics of that event. The recall accuracy seen in the present study relating to a convulsive‐type functional seizure may not generalize to other types of nonconvulsive functional seizures. Replication using different stimuli and in other populations would thus be valuable. However, consistency with prior work on epileptic seizures suggests that these findings may generalize across TLOC events. Prior experience of witnessing suspected seizures also varied across groups. Randomization was not stratified by this variable, and the observed differences likely reflect chance variation in modestly sized groups. Nevertheless, prior exposure may have influenced participants' recall of the video and should be considered when interpreting the findings.

Finally, we did not explicitly exclude health care professionals from participation or record participants' professions across conditions. The extent to which their inclusion may have influenced the generalizability of recall accuracy estimates overall or between groups is therefore unclear. Any effect may have been limited, as Mannan and Wieshmann^16^ found generally low accuracy of seizure descriptions and no consistently better recall among junior doctors or students with epilepsy teaching than among nonmedical participants in a small study of immediate recall following observation of an epileptic seizure video.

### 4.5 Conclusions

Eyewitness recall of an FDS is modest, declines over time in a manner consistent with established memory theory, and is not likely to be improved by early structured questioning. Witness confidence is a poor indicator of accuracy. Together with similar findings in epileptic seizures, these results suggest that reliance on eyewitness accounts in the diagnosis of TLOC should be cautious. Efforts to improve diagnostic accuracy should prioritize early information capture and explore complementary approaches beyond attempts to enhance recall.

## AUTHOR CONTRIBUTIONS

**Adam John Noble:** Conceptualization; methodology; formal analysis; investigation; resources; data curation; writing—original draft; project administration and supervision. **Steven Lane:** Methodology; formal analysis; data curation; writing—review & editing. **Amina Al‐Khaddar:** Investigation; methodology; writing—review & editing. **Annabelle Blundy:** Investigation; methodology; writing—review & editing. **Brenna Dubois:** Investigation; methodology; writing—review & editing. **Chloe Pearce:** Investigation; methodology; writing—review & editing. **Laszlo Sztriha**: Methodology; resources; writing—review & editing. **Graham Powell:** Methodology; resources; writing—review & editing. **Alistair Wardrope**: Methodology; resources; writing—review & editing. **Rajiv Mohanraj:** Methodology; resources; writing—review & editing. **Anthony Guy Marson:** Conceptualization; methodology; resources; writing—review & editing. **Markus Reuber:** Methodology; resources; writing—review & editing.

## CONFLICT OF INTEREST STATEMENT

None of the authors has any conflict of interests to disclose.

## ETHICAL PUBLICATION STATEMENT

We confirm that we have read the Journal's position on issues involved in ethical publication and affirm that this report is consistent with those guidelines. This study was approved by the University of Liverpool's Institute of Population Health Research Ethics Committee (Ref: 17266). All participants provided informed consent. The video used in the study has previously been published, but explicit written consent was secured from all persons featured in it for the purposes of the study.

## Supporting information


**APPENDIX S1.** Systematic literature search: Methods and summary of studies.
**FIGURE S1.** PRISMA (Preferred Reporting Items for Systematic Reviews and Meta‐Analyses) flowchart showing identification and selection assessment for updated to systematic literature search.
**TABLE S1.** Summary of findings from eligible studies identified by systematic search by Noble et al.^17^ and update.

**APPENDIX S2.** Additional details on recruitment process.

**APPENDIX S3.** Additional details on questions used to assess functional/dissociative seizure recall accuracy and how a definitive answer was determined.
**TABLE S3.1.** The 17 items from Erba et al.'s questionnaire initially presented to neurologists with answer options available to them.
**TABLE S3.2.** Experts' individual description of seizure in video according to 18 items from Erba et al.'s questionnaire and the extent of agreement.
**TABLE S3.3.** The final set of 15 items from Erba et al.'s questionnaire and answers used to evaluate participants' recall.

**APPENDIX S4.** Diagram of flow of participants through the study.

**APPENDIX S5.** The accuracy of recall participants in the individual conditions at different assessment points and their confidence.

## Data Availability

The data that support the findings of this study are available from the corresponding author upon reasonable request.
