# Supplementary material for: Do witnesses of functional/dissociative seizures recall them accurately? A UK experimental study of semiology recall at clinically relevant time points with a pilot intervention
Source: Epilepsia. 2026 Jul 10;67(10):5286–300. doi: 10.1002/epi.70388 (PMC13675776; doi:10.1002/epi.70388)
Supplement: Supplementary file 1 [file EPI-67-5286-s001.docx]

**Appendix S5** The accuracy of recall participants in the individual conditions at different assessment points and their confidence

|  | **CONDITION** | | | | | | | | | | | | | | | |
| --- | --- | --- | --- | --- | --- | --- | --- | --- | --- | --- | --- | --- | --- | --- | --- | --- |
|  | **A** | | | | **B** | | | | **C** | | | | **D** | | | |
|  | ***Baseline***  *(Free recall)*  (N=75) | | ***Follow-up***  *(2 weeks)*  (N=71) | | ***Baseline***  *(Free recall)*  (N=75) | | **Follow-up**  *(7 weeks)*  (N=64) | | **Baseline**  *(Systematic recall)*  (N=75) | | **Follow-up**  *(2 weeks)*  (N=66) | | **Baseline**  *(Systematic recall)*  N=75) | | **Follow-up**  *(7 weeks)*  (N=68) | |
| **Overall recall** | | | | | | | | | | | | | | | | |
| % Correct  Mean (SD)  Median (IQR)  Min-Max | n/a  n/a  n/a | | 61.3 (11.7)  60.0 (53.3-66.7)  40.0-86.7 | | n/a  n/a  n/a | | 53.4 (15.0)  53.3 (46.7-60.0)  13.3-80.0 | | 65.7 (13.7)  66.7 (60.0-73.3)  13.3-93.3 | | 56.1 (12.8)  53.3 (46.7-66.7)  13.3-80.0 | | 66.3 (12.8)  66.7 (60.0-73.3)  26.7-93.3 | | 53.3 (15.4)  53.3 (40.0-66.7)  20.0-93.3 | |
| Raw  Mean (SD)  Median (IQR)  Min-Max | n/a  n/a  n/a | | 9.2 (1.8)  9.0 (8.0-10.0)  6.0-13.0 | | n/a  n/a  n/a | | 8.0 (2.3)  8.0 (7.0-9.0)  2.0-12.0 | | 9.9 (2.1)  10.0 (9.0-11.0)  2.0-14.0 | | 8.4 (1.9)  8.0 (7.0-10.0)  2.0-12.0 | | 9.9 (1.9)  10.0 (9.0-11.0)  4.0-14.0 | | 8.0 (2.3)  8.0 (6.0-10.0)  3.0-14.0 | |
| **Individual recall items** | | | | | | | | | | | | | | | | |
|  | ***Correct n (%)*** | ***Rank*** | ***Correct***  ***n (%)*** | ***Rank^a^*** | ***Correct n (%)*** | ***Rank*** | ***Correct***  ***n (%)*** | ***Rank^a^*** | ***Correct***  ***n (%)*** | ***Rank^a^*** | ***Correct***  ***n (%)*** | ***Rank^a^*** | ***Correct***  ***n (%)*** | ***Rank^a^*** | ***Correct***  ***n (%)*** | ***Rank^a^*** |
| 1. Did you observe any of the following at the very beginning of the seizure? | n/a | n/a | 46 (64.8) | =6 | n/a | n/a | 40 (62.5) | 6 | 47 (62.7) | =8 | 34 (51.5) | =8 | 43 (57.3) | 12 | 31 (45.6) | 9 |
| 2. Did the patient shake or stiffen? | n/a | n/a | 70 (98.6) | 1 | n/a | n/a | 58 (90.6) | 1 | 73 (97.3) | =1 | 63 (95.5) | 2 | 75 (100) | 1 | 65 (95.6) | 1 |
| 3. How did the shaking or stiffening start? | n/a | n/a | 40 (56.3) | 9 | n/a | n/a | 28 (43.8) | 8 | 45 (60.0) | =9 | 26 (39.4) | 11 | 54 (72.0) | =6 | 28 (41.2) | 11 |
| 4. How did the patient shake or stiffen? | n/a | n/a | 45 (63.4) | 8 | n/a | n/a | 39 (60.9) | 7 | 49 (65.3) | =6 | 34 (51.5) | =8 | 54 (72.0) | =6 | 35 (51.5) | 7 |
| 5. Did the shaking or stiffening stop abruptly and then start back up during the seizure? | n/a | n/a | 53 (74.6) | 5 | n/a | n/a | 44 (68.8) | 5 | 59 (78.7) | 5 | 53 (80.3) | =5 | 61 (81.3) | 5 | 55 (80.9) | 4 |
| 6. How did the shaking or stiffening stop? | n/a | n/a | 34 (47.9) | 10 | n/a | n/a | 16 (25.0) | 13 | 33 (44.0) | 11 | 24 (36.4) | 12 | 23 (30.7) | 13 | 12 (17.6) | 14 |
| 7. Did the patient’s head turn strongly to one side? | n/a | n/a | 23 (32.4) | 13 | n/a | n/a | 17 (26.6) | 12 | 45 (60.0) | =9 | 25 (37.9) | 13 | 43 (57.3) | =8 | 24 (35.3) | 12 |
| 8. Was there movement of the head from side to side? | n/a | n/a | 43 (60.6) | 11 | n/a | n/a | 21 (32.8) | 9 | 62 (82.7) | 4 | 36 (54.5) | 7 | 65 (86.7) | 4 | 33 (48.5) | 6 |
| 9. During the seizure were the patient’s eyes closed or open? | n/a | n/a | 10 (14.1) | 15 | n/a | n/a | 9 (14.1) | 15 | 4 (5.3) | 15 | 6 (9.1) | 15 | 4 (5.3) | 15 | 4 (5.9) | 15 |
| 10. Did you see any of the following during the seizure? | n/a | n/a | 33 (46.5) | 12 | n/a | n/a | 18 (28.1) | =10 | 30 (40.0) | 13 | 20 (30.3) | 14 | 37 (49.5) | 10 | 30 (44.1) | 10 |
| 11. Do you see any of the following during the seizure? | n/a | n/a | 21 (29.6) | 14 | n/a | n/a | 18 (28.1) | =10 | 31 (41.3) | 12 | 23 (34.8) | 13 | 27 (36.0) | 12 | 21 (30.9) | 13 |
| 12. Did the patient fall during the seizure? | n/a | n/a | 69 (97.2) | 2 | n/a | n/a | 55 (85.9) | 2 | 73 (97.3) | =1 | 64 (97.0) | 1 | 74 (98.7) | 2 | 64 (94.1) | 2 |
| 13. How long did the actual seizure activity last? | n/a | n/a | 58 (81.7) | 4 | n/a | n/a | 49 (76.6) | 4 | 71 (94.7) | 3 | 53 (80.3) | =5 | 70 (93.3) | =3 | 52 (76.5) | 5 |
| 14. Right at the end of the seizure activity, did you see any of the following? | n/a | n/a | 46 (64.8) | =6 | n/a | n/a | 48 (75.0) | 3 | 49 (65.3) | =6 | 37 (56.1) | 6 | 46 (61.3) | 7 | 32 (47.1) | 8 |
| 15. Right at the end of the seizure activity, what was their level of consciousness and awareness? | n/a | n/a | 62 (87.3) | 3 | n/a | n/a | 53 (82.8) | 3 | 68 (90.7) | 2 | 57 (86.4) | 3 | 70 (93.3) | =3 | 58 (85.3) | 3 |
| **CONFIDENCE IN RECALL** | | | | | | | | | | | | | | | | |
| Mean (SD) | n/a | | 5.82 (1.30) | | n/a | | 4.64 (1.72) | | n/a | | 5.68 (1.27) | | n/a | | 5.31 (1.30) | |

***Notes:*** n, number; n/a, not applicable as participants in this condition were not asked to complete this measure. IQR, interquartile range; Min, minimum; Max, maximum; SD, standard deviation.

^a^ Questions for each recall item were ranked for each condition/assessment point according to the proportion of correct responses elicited (with rank 1= highest proportion of correct responses).
