# Supplementary material for: Do witnesses of functional/dissociative seizures recall them accurately? A UK experimental study of semiology recall at clinically relevant time points with a pilot intervention
Source: Epilepsia. 2026 Jul 10;67(10):5286–300. doi: 10.1002/epi.70388 (PMC13675776; doi:10.1002/epi.70388)
Supplement: Supplementary file 2 [file EPI-67-5286-s002.docx]

**Appendix S4**	Diagram of flow of participants through the study

***Notes:*** Condition A, Immediate free-recall, 2-week follow-up; B, Immediate free-recall, 7-week follow-up; C, Immediate systematic recall, 2-week follow-up; D, Immediate systematic recall, follow-up 7 weeks.
