# Supplementary material for: Do witnesses of functional/dissociative seizures recall them accurately? A UK experimental study of semiology recall at clinically relevant time points with a pilot intervention
Source: Epilepsia. 2026 Jul 10;67(10):5286–300. doi: 10.1002/epi.70388 (PMC13675776; doi:10.1002/epi.70388)
Supplement: Supplementary file 4 [file EPI-67-5286-s004.docx]

**Appendix S1**	Systematic literature search: Methods and summary of studies

**Orientation**

Noble et al. ^1^ conducted a systematic search of Scopus, MEDLINE, and Web of Science from database inception to 29 September 2024 to identify evidence on the accuracy of first-time witnesses of suspected seizures. Three studies reported on ‘first time’ witness recall of epileptic seizures (Table S1). No studies had examined recall of functional/dissociative seizures (FDS). We updated the search to identify any additional studies published since that date on recall across all seizure types.

**Methods**

*Identification*

A search of Scopus, Medline and Wed of Science was conducted for evidence published between 30/10/2024 and 27/10/2025  using the same search terms (article titles and abstracts), modified for the different databases:

1. witness OR bystander OR informant OR caller
2. AND recall OR memory OR testimony OR remember OR account
3. AND seiz* OR epilep* OR convuls* OR "non epileptic attack" OR "NEAD" OR "functional neurological disorder" OR "FND" OR "loss of consciousness"

*Eligibility and screening*

To be eligible an article had to be reported in English, be published in a peer-reviewed journal and the study needed to have examined the accuracy of ‘first time’ witness recall of a suspected seizure.

Titles/abstracts for identified articles were screened for eligibility by two reviewers (AN, BD), removing duplicates and obviously irrelevant studies. Full texts versions of the articles that they both agreed as ostensibly eligible were accessed and further reviewed for eligibility by both reviewers. There was complete agreement between the reviewers, with no discrepancies to resolve.

**Results**

The selection process and reasons for exclusion are shown in the PRISMA flow chart Figure S1 below. Ultimately 1 eligible study – namely, that conducted by our group ^1^ which had itself conducted the prior systematic literature search – was identified. It is summarised in the Table S1. It examined witness recall immediately after watching a video of epileptic seizure. No evidence was presented on witness recall of FDS.

Total identified (n = 19)

Web of Science
(n = 4)

Medline
(n = 7)

Scopus

(n=8)

Duplicate records (n =7)

Unique records

(n = 12)

Records excluded based on title and abstract
(n =10)

*Reasons:* Recall not tested= 10

Full text articles examined

(n = 2)

Records excluded after full article review

(n =1)

*Reasons:* Not in English= 1

Eligible studies identified

(n = 1)

**FIGURE S1**	PRISMA Flow chart showing identification and selection assessment for updated to systematic literature search

**TABLE S1**	Summary of findings from eligible studies identified by systematic search by Noble et al.^1^ and update

| **Identified by** | **Author** | **Population** | **Event observed** | **Sample size** | **Recall timing** | **Accuracy assessment method** | **Overall accuracy** | **Key correctly recalled items** | **Common errors** | **Notes** |
| --- | --- | --- | --- | --- | --- | --- | --- | --- | --- | --- |
| Noble et al. original search^1^ | Mannan & Wieshmann ^2^ | Medical/non-medical students and junior doctors (England) | Video of focal seizure that generalized.  Played with advanced warning. | 20 | Immediately after viewing. | Written accounts scored by 2 neurologists against eight semiological features.  One point for each correct item; one deduced for each incorrect observation | Mean score: 2.7 (range −2 to 6) out of 8. | - | Misinterpretations (e.g., 'rolled over', 'agitated').    Unresponsiveness and lateralizing signs often missed. | Prior epilepsy teaching had no consistent effect.  No attention-check embedded in assessment. |
| Noble et al. original search^1^ | Thijs et al.^3^ | Psychology students (Netherlands) | Video of generalized tonic–clonic seizure or reflex syncope.  Played *without* advanced warning. | 229 | Immediately after viewing. | 11 study-specific questions scored against consensus view of 4 neurologists. | Seizure: On items observable in the video, 60% of responses were correct. Syncope: 44% correct. | - | - | No effect of sex, prior experience, or question order.  Attention check embedded in assessment. |
| Noble et al. original search^1^ | Muayqil et al.^4^ | General population adults  (Saudi Arabia) | Video of generalizing or non-generalizing seizure.  Played with advanced warning. | 172 | Immediately after viewing. | Interview-administered semiology questions scored against consensus view of 3 neurologists.  17 questions for generalizing seizure, and 13 for non-generalizing seizure. | Generalizing: 64.7% correct.  Non-generalizing: 54.7% correct | Generalizing: ‘Involvement of head’ (89.9%) and involvement of an arm (84.8%).  Non-generalizing: ‘Involvement of head’ (93.8%) and whether there was a focal start to the seizure (88.8%). | Generalizing: Errors with vertical head movement and presence of vocalization.  Non-generalizing: Which leg/s moved and type of leg movement. | No attention-check embedded in assessment. |
| Updated search | Noble et al.^1^ | General population adults (Unted Kingdom) | Video of a focal‐to‐bilateral tonic–clonic epileptic seizure.  Played with advanced warning. | 304 | Participants randomised to different recall intervals that included ‘immediately’ after viewing (following a 9‐min filler task designed to simulate ambulance response time), 2-weeks later, and 7-weeks later. | 15 semiology questions derived from a standardised questionnaire scored against consensus view of 5 neurologists. | Participants answered a mean of 67% of questions correctly immediately after viewing the seizure, 54% at 2 weeks and 50% at 7 weeks. | Recall gross of motor features (e.g., shaking, falling) was most accurate. | Items more poorly recalled included finer or more abstract features (e.g., shaking onset, awareness).  Features sometimes considered diagnostically valuable—such as ictal eye closure or side‐to‐side head movements—were poorly recalled, with typically <50% accuracy at 2 and 7 week follow-up. | Witness confidence in their recall did not reliably indicate how accurate their recall was.  Systematic questioning of witnesses immediately after seeing the seizure improved later recall by up to 10%. |

**REFERENCES**

1.	Noble AJ, Lane S, New P, Cope H, Foley C, Williams HL, et al. How accurate are witnesses of first suspected seizures in recalling semiology at clinically relevant timepoints? A UK experimental study with a pilot intervention Epilepsia. 2025 Dec;66:4795-4808.

2.	Mannan JB, Wieshmann UC. How accurate are witness descriptions of epileptic seizures? Seizure. 2003 Oct;12:444-447.

3.	Thijs RD, Wagenaar WA, Middelkoop HA, Wieling W, van Dijk JG. Transient loss of consciousness through the eyes of a witness Neurology. 2008 Nov 18;71:1713-1718.

4.	Muayqil TA, Alanazy MH, Almalak HM, Alsalman HK, Abdulfattah FW, Aldraihem AI, et al. Accuracy of seizure semiology obtained from first-time seizure witnesses BMC Neurol. 2018 Sep 1;18:135.
