# Supplementary material for: Do witnesses of functional/dissociative seizures recall them accurately? A UK experimental study of semiology recall at clinically relevant time points with a pilot intervention
Source: Epilepsia. 2026 Jul 10;67(10):5286–300. doi: 10.1002/epi.70388 (PMC13675776; doi:10.1002/epi.70388)
Supplement: Supplementary file 5 [file EPI-67-5286-s005.docx]

**Appendix S3** Additional details on questions used to assess functional/dissociative seizure

recall accuracy and how a definitive answer was determined

A total 17 questions from Erba et al.’s ^1^ seizure witness questionnaire which could plausibly be answered by someone watching a video of stranger were identified to form the basis of the recall accuracy outcome measure for the study. The list of questions is shown in Table S3.1. Items relating to obvious internal experiences of the patient were not included (e.g., "Does the patient report a change in feeling or no sensation during the seizure?").

To establish a ‘gold standard’ description of the presentation depicted in the video according to the 17 questions, 5 UK based consultant neurologists with expertise in seizures (as defined by NICE criteria ^2^) separately viewed the video and were presented with the questions and asked to provide their responses based on their professional judgment. This is a similar process to that employed by previous studies of seizure witness recall.^3-5^

Table S3.2 shows the neurologists individual answers to each of the items. One ‘responsive’ question relating to *how* a patient fell was ultimately not asked because none of the neurologists reported, according to a preceding question, that the patient had fallen. For one item, relating to how did the patient’s shaking or stiffening started, the neurologists formed no consensus or majority view. For the remaining 15 questions, the neurologists were in complete consensus (n=7) or there was a majority view (n=8). We used these 15 items in the study to evaluate participants recall accuracy. Table S3.3 provides the questions, and the answers used to evaluate participants recall.

**Table S3.1** The 17 items from Erba et al.’s questionnaire initially presented to neurologists with answer options available to them

| **Question** | **Answer Options** |
| --- | --- |
| a) Did you observe any of the following at the very beginning of the seizure? (CHECK ALL THAT APPLY) | • stopping and staring • high-pitched cry • humming or other vocalization • none of these |
| b) Did the patient shake or stiffen? (CHECK ONE ONLY) | • no • yes • cannot say/unsure |
| *If yes to b),*   c) How did the shaking or stiffening start? (CHECK ONE ONLY) | • suddenly • built up gradually • cannot say/unsure |
| *If yes to b),*   d) How did the patient shake or stiffen? (CHECK ONE ONLY) | • on one side only • on both sides equally • from one side to the other during the seizure • cannot say/unsure |
| *If yes to b),*   e) Did the shaking or stiffening stop abruptly and then start back up during the seizure? (CHECK ONE ONLY) | • no • yes • cannot say/unsure |
| *If yes to b),*   f) How did the shaking or stiffening stop? (CHECK ONE ONLY) | • suddenly • slowed down gradually • cannot say/unsure |
| g) Did the patient’s head turn strongly to one side? (CHECK ONE ONLY) | • no • yes • cannot say/unsure |
| h) Was there movement of the head from side to side? (CHECK ONE ONLY) | • no • yes • cannot say/unsure |
| i) During the seizure, were the patient’s eyes closed or open? (CHECK ONE ONLY) | • closed • open • cannot say/unsure |
| j) Did you see any of the following during the seizure? (CHECK ALL THAT APPLY) | • staring • wandering around • picking at things • one arm bent and the other arm stretched out • sudden, brief jerks of arms or legs • agitated behaviour • making loud noises • sobbing or crying • none of these |
| k) Do you see any of the following during the seizure? (CHECK ALL THAT APPLY) | • thrashing or 'flopping like a fish' • limb movements which were 'out of sync' • back arching • hip thrusting • none of these |
| l) How was the patient’s breathing during the seizure? (CHECK ONE ONLY) | • normal • fast and heavy • not breathing, may have turned blue or grey • cannot say/unsure |
| m) Did the patient fall during the seizure? (CHECK ONE ONLY) | • no • yes • cannot say/unsure |
| If yes to m),   n) What did the fall look like? (CHECK ONE ONLY) | • slow, gradual slumping • sudden collapse or drop • sudden stiffening and toppling over • cannot say/unsure |
| o) How long did the actual seizure activity last? (CHECK ONE ONLY) | • less than 2 minutes • 2-10 minutes • > 10 minutes • cannot say/unsure |
| p) Right at the end of the seizure activity, did you see any of the following? (CHECK ALL THAT APPLY) | • cough • wipe their nose • breathing was noisy and congested • none of these |
| q) Right at the end of the seizure activity, what was their level of consciousness and awareness? (CHECK ONE ONLY) | • asleep • awake but confused and disoriented • normal • cannot say/unsure |

**TABLE S3.2**	Experts individual description of seizure in video according to 18 items from Erba et al.’s questionnaire and the extent of agreement

| **Question** | **Expert - consultant neurologist - raters** | | | | | **Complete**  **consensus?** | **If not, a majority view?** | **Consensus answer taken for study** |
| --- | --- | --- | --- | --- | --- | --- | --- | --- |
|  | ***Rater #1*** | ***Rater #2*** | ***Rater #3*** | ***Rater #4*** | ***Rater #5*** |  |  |  |
| a) Did you observe any of the following at the very beginning of the seizure? | Stopping and staring | None of these | None of these | None of these | None of these | No | *Yes* | None of these |
| b) Did the patient shake or stiffen? | Yes | Yes | Yes | Yes | Yes | *Yes* | *n/a* | Yes |
| c) How did the shaking or stiffening start? | Cannot say/ unsure | Suddenly | Cannot say/ unsure | Suddenly | Built up gradually | No | No | *n/a* |
| d) How did the patient shake or stiffen? | From one side to the other during the seizure | On both sides equally | On both sides equally | On both sides equally | On both sides equally | No | *Yes* | On both sides equally |
| e) Did the shaking or stiffening stop abruptly and then start back up during the seizure? | Yes | Yes | No | Yes | No | No | *Yes* | *Yes* |
| f) How did the shaking or stiffening stop? | Suddenly | Slowed down gradually | Slowed down gradually | Slowed down gradually | Slowed down gradually | No | *Yes* | *Slowed down gradually* |
| g) Did the patient’s head turn strongly to one side? | No | No | No | No | No | *Yes* | *n/a* | *No* |
| h) Was there movement of the head from side to side? | Yes | Yes | Yes | Yes | Yes | *Yes* | *n/a* | *Yes* |
| i) During the seizure were the patient’s eyes closed or open? | Closed | Closed | Closed | Closed | Closed | *Yes* | *n/a* | *Closed* |
| j) Did you see any of the following during the seizure? | None of these | None of these | One arm bent and the other arm stretched out | One arm bent and the other arm stretched out | None of these | No | *Yes* | None of these |
| k) Did you see any of the following during the seizure? | Limb movements which were 'out of sync' | Limb movements which were 'out of sync' | Limb movements which were 'out of sync' | Limb movements which were 'out of sync' | Limb movements which were 'out of sync' | *Yes* | *n/a* | *Limb movements which were 'out of sync'* |
| l) How was the patient’s breathing during the seizure? | Normal | Normal | Cannot say/ unsure | Cannot say/ unsure | Normal | No | *Yes* | *Normal* |
| m) Did the patient fall during the seizure? | No | No | No | No | No | *Yes* | *n/a* | *No* |
| n) What did the fall look like? | Not asked as responsive question | Not asked as responsive question | Not asked as responsive question | Not asked as responsive question | Not asked as responsive question | *Not asked due to answer to prior question* | *n/a* | *n/a* |
| o) How long did the actual seizure activity last? | 2-10 minutes | 2-10 minutes | 2-10 minutes | Less than 2 minutes | 2-10 minutes | No | *Yes* | *2-10 minutes* |
| p) Right at the end of the seizure activity, did you see any of the following? | None of these | None of these | None of these | None of these | None of these | *Yes* | *n/a* | *None of these* |
| q) Right at the end of the seizure activity, what was their level of consciousness and awareness? | Awake but confused and disoriented | Cannot say/ unsure | Awake but confused and disoriented | Awake but confused and disoriented | Cannot say/ unsure | No | *Yes* | *Awake but confused and disoriented* |

**TABLE S3.3**	The final set of 15 items from Erba et al.’s questionnaire and answers used to evaluate participants recall

| **Individual questions** | **Correct answer** |
| --- | --- |
| 1. **Did you observe any of the following at the very beginning of the seizure? (check all that apply)**   Options: • stopping and staring; • high-pitched cry; • humming or other vocalization; • none of these | *None of these* |
| 2. **Did the patient shake or stiffen? (check one only)**   Options: • no; • yes; • cannot say/unsure | *Yes* |
| 3. **How did the patient shake or stiffen? (check one only)** ^a^   Options: • on one side only; • on both sides equally; • from one side to the other during the seizure; • cannot say/unsure | *On both sides equally* |
| 4. **Did the shaking or stiffening stop abruptly and then start back up during the seizure? (check one only)** ^a^   Options: • no; • yes; • cannot say/unsure | *Yes* |
| 5. **How did the shaking or stiffening stop? (check one only)** ^a^   Options: • suddenly; • slowed down gradually; • cannot say/ unsure | *Slowed down gradually* |
| 6. **Did the patient’s head turn strongly to one side? (check one only)**   Options: • no; • yes; • cannot say/ unsure | *No* |
| 7. **Was there movement of the head from side to side? (check one only)**   Options: • no; • yes; • cannot say/ unsure | *Yes* |
| 8. **During the seizure were the patient’s eyes closed or open? (check one only)**   Options: • closed; • open; • cannot say/unsure | *Closed* |
| 9. **Did you see any of the following during the seizure? (check all that apply)**   Options: • staring; • wandering around; • picking at things; • one arm bent and the other arm stretched out; • sudden, brief jerks of arms or legs; • agitated behaviour; • making loud noises; • sobbing or crying; • none of these | *None of these* |
| 10. **Do you see any of the following during the seizure? (check all that apply)**   Options: • thrashing or 'flopping like a fish'; • limb movements which were 'out of sync'; • back arching; • hip thrusting; • none of these | *Limb movements which were 'out of sync'*  (Note - No other options can be selected in addition to the above. If any other options are selected in addition to the above this would render the person’s answer incorrect) |
| 11. **How was the patient’s breathing during the seizure? (check one only)**   Options: • normal; • fast and heavy; • not breathing, may have turned blue or grey; • cannot say/unsure | *Normal* |
| 12. **Did the patient fall during the seizure? (check one only)**   Options: • no; • yes; • cannot say/ unsure | *No* |
| 13. **How long did the actual seizure activity last? (check one only)**   Options: • less than 2 minutes; • 2-10 minutes; • > 10 minutes; • cannot say/ unsure | *2-10 minutes* |
| 14. **Right at the end of the seizure activity, did you see any of the following? (check all that apply)**   Options: • cough; • wipe their nose; • breathing was noisy and congested; • none of these | *None of these* |
| 15. **Right at the end of the seizure activity, what was their level of consciousness and awareness?** **(check one only)**   Options: • asleep; • awake but confused and disoriented; • normal; • cannot say/unsure | *Awake but confused and disoriented* |

***Notes:*** ^a^ If person answered incorrectly to Q2 that the person did not shake, they were not asked Qs 3-5 and thus judged automatically incorrect on these items for scoring purposes.

**REFERENCES**

1.	Erba G, Bianchi E, Giussani G, Langfitt J, Juersivich A, Beghi E. Patients' and caregivers' contributions for differentiating epileptic from psychogenic nonepileptic seizures. Value and limitations of self-reporting questionnaires: A pilot study Seizure. 2017 Dec;53:66-71.

2.	National Institute for Health and Care Excellence. Epilepsies in children, young people and adults: Quality standard. Available at: <https://www.nice.org.uk/guidance/qs211>. Accessed 2 April.

3.	Muayqil TA, Alanazy MH, Almalak HM, Alsalman HK, Abdulfattah FW, Aldraihem AI, et al. Accuracy of seizure semiology obtained from first-time seizure witnesses BMC Neurol. 2018 Sep 1;18:135.

4.	Noble AJ, Lane S, New P, Cope H, Foley C, Williams HL, et al. How accurate are witnesses of first suspected seizures in recalling semiology at clinically relevant timepoints? A UK experimental study with a pilot intervention Epilepsia. 2025 Dec;66:4795-4808.

5.	Thijs RD, Wagenaar WA, Middelkoop HA, Wieling W, van Dijk JG. Transient loss of consciousness through the eyes of a witness Neurology. 2008 Nov 18;71:1713-1718.
